# Surgery in the Next Space Missions

**DOI:** 10.3390/life13071477

**Published:** 2023-06-29

**Authors:** Desiree Pantalone

**Affiliations:** 1 American College of Surgeons, FACS, Chicago, IL 60611, USA; 2 Department of Experimental and Clinical Medicine, University of Florence, 50134 Florence, Italy; desire.pantalone@unifi.it; Tel.: +39-335-677-5724; 3 Emergency Surgery Unit-Trauma Team, Trauma Center, Careggi University Hospital, 50134 Florence, Italy; pantaloned@aou-careggi.toscana.it; Tel.: +39-055-794-7009

**Keywords:** space research, trauma, human physiology in space, robotic surgery, Mars, deep-space missions

## Abstract

In the coming years, missions to the Moon and Mars shall be the new goals of space flight. The complexity of these missions due to the great distance from Earth and the unforeseen obstacles to settle on another planet have given rise to great concerns for crew health and survival. The need for advanced crew autonomy and a different approach to surgical emergency require new protocols and devices to help future crew medical officers and other crew members in a task of unprecedented difficulty. Hence, the increasing variety of schedules, devices, and protocols being developed. A serious health problem, such as an emerging surgical disease or severe trauma, can jeopardize the mission and survival of the entire crew. Many other difficulties are present in deep-space missions or settlements on other planets, such as communication and supply, also medical, delays, and shortage, and the presence of radiation. Progress in advanced technologies as well as the evolution of robotic surgery and the use of artificial intelligence are other topics of this review. In this particular area of research, even if we are still very far from an “intelligent robot”, this evolution must be evaluated in the light of legislative and ethical considerations. This topic was presented at the annual meeting of the American College of Surgeons—Italy Chapter in 2021.

## 1. Introduction

Space research has undergone many changes through time. Today, the International Space Station (ISS) is no longer the exclusive target as space flight is destined to go beyond the limit of the Earth’s atmosphere [1,2], thus making space orbital stations, capable of working with or without a crew, the new frontiers. Similar advances toward increased crew autonomy were achieved in the approach to surgical emergencies based on the different types of missions. The policy of a rapid return to Earth, currently adopted on the ISS in low Earth orbit (LEO), shall no longer be the chosen option in case of an emergency surgical operation. In fact, during lunar missions the distance from Earth and the Gateway orbit, may lengthen up to two weeks the time for the return to Earth [3,4]. Increased levels of crew autonomy in medical issues shall be necessary also in exploratory-class missions or after landing on Mars [5,6].

The effects on human physiology, caused by launch-related hypergravity, absence of the effects of gravity in space [5,6], lack of radiation protection outside the Earth geomagnetic field [7,8], and gravitational conditions, must also be taken into account when landing on another planet after a long voyage in space [5,6,7,8].

In space missions, numerous health changes occur as a result of these peculiar conditions, but equally important are including possible effects on mental health caused by confinement, limited privacy, or lack of physical activity (walks) during exploratory-class missions. The effects caused by the absence of gravity on human physiology are numerous and the consequences of longer stays in space (four to five years), compared to current missions, have not been studied yet [5,6,7,8]. In these types of missions, ground support will be progressively hampered by the increasing distances from the Earth as will communications due to the limit imposed by the speed of light causing a signal delay [9,10]. Also, opportunities of resupply and any kind of aid, presently possible on the ISS in LEO, shall decrease with the growing distance from Earth. This narrative review aims at exploring the state of the art of space-surgery research and the increased levels of crew autonomy required for future missions to other planets or in deep space. These challenges pave the way for the application of artificial-intelligence technology, the miniaturization of medical and surgical devices, the use of 3D printers and other new achievements that shall enable the crew to be increasingly more self-sufficient and be prepared to face unforeseen risks and health problems arising when exploring distant and hostile environments. Particular attention will be dedicated to the possibility of a surgical smart robot aboard. A robot capable of autonomously performing some task to relieve human fatigue is an intriguing possibility from which we are still very far. Panesar et al. [11] in their publication estimate that this solution could be, perhaps, realized only at the end of this century due to the necessary technology improvements that need to be made. Also needed is a careful definition of what may be the tasks that a surgical smart robot should be allowed to do, surgery being a peculiarity of humans not only for its technical complexity but also for all the legislative and moral aspects [11].

## 2. Materials and Methods

The research for publications was conducted on MEDLINE, PubMed, and Google Scholar. Relevant publications were identified using the terms “space surgery”, “microgravity”, “zero gravity”, “missions to Mars”, and “exploratory-class missions”. The selected articles must be in English or provided with an English exhaustive abstract, if in another language. A search was conducted to identify relevant publications. The site of the European Space Agency (ESA) and the National Aeronautics and Space Administration (NASA) were examined to find information on the next space missions.

## 3. Space Missions Overview

In a paper of 2016 by Komorowsky et al. [12,13], the design concept for missions to Mars corresponds to a six-person crew conducting a 900-day mission. Recently, NASA has been developing plans for “deep space” cislunar missions in the 2020s, followed by Mars missions in the 2030s, for “at least 4 crew members” lasting “up to 1100 days” [13,14]. In missions to the Moon or Mars, the duration, remoteness, and type of activities lead to hazards not present in ISS missions, such as exposure to reduced gravity (about one sixth of Earth’s gravity on the Moon and one third on Mars), ionizing radiations, meteoroids, planetary dust, hypobaric decompression sickness, and extreme temperatures [15,16,17]. Another increased risk is related to extravehicular activities during surface exploration that will expose astronauts to a higher level of possible traumatic events and hypobaric-decompression sickness [7,16,17,18,19,20,21]. In addition, bone-density reduction due to exposure to weightlessness and to partial gravity, leads to high osteoporotic levels that, after a few months, exposes astronauts to increased risk of pathologic fractures if no countermeasures are adopted [22].

Also in deep space, the required level of crew autonomy in medical care will be exceptionally high [23,24]. An astronaut survey has, indeed, recommended having a medical doctor with broad medical skills on board as the best option [14,23,24]. The environmental control and life-support systems (ECLSS) in the spacecraft support human life by maintaining a stable, normoxic and normobaric atmosphere, with neutral temperatures and average concentrations of carbon dioxide 10 times higher than on the ground (0.3–0.5%). Moreover, space, with its nil barometric pressure, high levels of radiation, and extreme temperatures (−150 °C to 120 °C) [13], is, by definition, considered as “one of the most extreme environments imaginable” [25,26]. A sudden loss in cabin pressure can trigger an immediate life-threatening hazard (for example, if a meteor or satellite debris hits the spacecraft). Intra-vehicular flammable materials, potentially explosive devices, toxic gas and radiation sources, or ECLSS failure are other possible risk factors [22,26].

Given that missions to another planet or long-lasting voyages in deep space are inherently risky, massive effort will need to be made to maximize crew health and performance together with vehicle design and optimization of resource storage [26].

Miniaturized medical devices enabling the use of natural orifice transluminal endoscopic surgery (NOTES) and other minimally invasive surgeries are already under study today, with a robotic surgery simulation being scheduled for 2024 on the International Space Station. Although these miniaturized devices could be perfect for spacecrafts conceived for long-duration missions, which are inaccessible to the bulky surgical robots currently in use on Earth, it is worth emphasizing once again that studies of any type of intelligent robot are still in their infancy and, presently, no surgical robot is capable of full autonomy [27,28].

## 4. Autonomous Medical Care and the Crew

### 4.1. Surgery in Space: From Parabolic Flight Experiments to Surgical Robot Autonomy

Surgery in space has been studied from the beginning of space flights, mainly during parabolic flights of animal models [29]. Parabolic flights started in 1950, reproducing short gravity-free periods of up to 30 s. From the 1960s, bigger aircraft have been used for experiments in different disciplines [30,31]. Presently, they are used to prepare space-missions research, providing investigators with a test laboratory for scientific experimentation where gravity levels change repeatedly, alternating periods of reduced gravity with periods of hypergravity [32,33]. A variety of experiments with animal models have been performed but also human physiology in microgravity prior to, or after, a space mission has been investigated. For surgery procedures, restraint devices for operators and patient, surgical instruments, and supplies have been tested as well [31,33,34] (STS-90 Neurolab shuttle mission 1998 [34]). In 2005, Campbell et al. [32,33] wrote a publication about the experiments on rats during the 16 d STS-90 Neurolab mission. The surgical procedures included thoracotomies, laparotomies, craniotomies, laminectomies, and exposure of lower-extremity muscle and were performed under general anaesthesia through intramuscular and intraperitoneal injection within the general-purpose workstation (GPWS) on board the Columbia shuttle. A contained glovebox that enclosed the animals and surgical hardware for isolation from the spacecraft atmosphere was used. The dissection table had animal restraints attached with Velcro to the GPWS floor along with customized kits of surgical instruments and preloaded syringes of anaesthetics. All the devices were designed expressly for the experiments and also included sharps-containment procedures and disposal containers. Biohazard trash was properly stored in airtight containers and the GPWS had a closed air-circulation loop with an activated charcoal canister for odour control and to prevent organic contamination during the procedures.

A well-defined method of restraint was also designed for operators, animals, as well as surgical instruments by means of Velcro strips, elastic bands and individual restraint compartments.

A 2 y training period was required before the flight, and procedures were developed, evaluated, and verified on the ground and then in KC-135 parabolic flights (KC-135) [31].

Many results were obtained from these experiments, most of which required a high level of surgical skills. The preflight parabolic simulations appeared equally difficult and complex to participants and the procedures appeared comparable to those performed during spaceflight.

Likewise, no significant changes were detected by crewmembers in dexterity, fine-motor control, and proprioception in dissections performed during the 16-day mission. In particular, no differences were found in their surgical performance after acclimatization compared to flight experiences at 1 g. Even delicate surgical procedures did not appear to be any more difficult in 0 g spaceflight than in 1 g training sessions. However, the need to keep operators, animals, and instruments restrained led to an extension of the operating times of 1.5 to 2.

Conclusions were that further investigation is required to define the wound-healing process in space and pave the way for future exploration initiatives beyond low Earth orbit. Results proved that the execution of demanding surgical manoeuvres requiring advanced technical skills is feasible in space although not enough is known yet about the behaviour of human physiology in the case of adverse occurrences requiring major surgery [35,36]. In particular, the anaesthesiologic aspects need to be better studied and assessed due to the insufficient information so far available [17,31].

Previous experiments, such as STS-58 Spacelab Life Science (SLS) [34] shuttle mission 1993 and STS-90 Neurolab shuttle mission 1998 [34], tested surgical procedures on animals and changes in their central nervous system during microgravity [32].

The evolution of surgical techniques then led to the use of minimally invasive surgery (MIS, laparoscopic surgery) [32,37,38,39] and robotic-assisted mini-invasive surgery (RAMIS) [32,37,38,39] with the aid of telementoring or by teleoperating the surgical robot from a location on Earth in a specific NASA project known as NEEMO [40,41,42,43] that deployed the Aquarius underwater laboratory, the only undersea research station in the world, located 5.6 km off the coast in the Florida National Marine Sanctuary [40,41,42,43].

However, as spaceships will travel longer distances from Earth the biggest challenge remains the increasingly greater autonomy that will be needed to perform space surgery. This evolution involves a different approach to crew health and events that may require surgery. None of the options currently available on Earth—properly equipped facilities with operating rooms, trained personnel, diagnostic tools, blood supply, or intensive care units—are feasible on spaceships or in new settlements on another planet. This condition requires careful planning and crew risk-assessment prior to launch. Space missions must be managed similar to Earth missions in extreme environments (missions in Antarctica, for example) [44], although incoming missions in space destined to the exploration of other planets, ref. [15], have, compared to them, higher probability of trauma, haemorrhagic shock, and infections [45,46,47]. Also, Moon exploration missions and missions to Mars, whose goal is to establish a permanent settlement, lead to exposure to quite different hazards from the present conditions on the ISS in low Earth orbit. The extravehicular activities for surface exploration will place astronauts at a higher risk of traumatic accidents and hypobaric-decompression sickness [15,17]. In a publication of 2021, Komorowsky et al. [17] explored the literature about the peculiar conditions that crew members will have to face in the new missions, such as medical evacuation from deep space, issues related to supplies and refurbishment, and the abilities that will be required for any life-threatening events that may arise on board and requiring medical and surgical management. The latter will also be caused by changes on human physiology and the need to tailor the eventual use of anaesthetic techniques to a number of factors, such as patient conditions, individual experience, availability of drugs and equipment, and the need to act in an emergency setting. So far, no adverse event requiring surgery has ever occurred, albeit the worst scenario usually considered is the patient being severely deconditioned, hypovolemic, a difficult intubation, or drug intolerance; also worthy of mention are other conditions, such as a full stomach, and the unavailability of trained and experienced personnel. Also, to date, the variety of possible anaesthetic techniques has not been completely explored [16,17].

The concept of surgery in space has evolved to laparoscopic mini-invasive surgery (MIS) [48,49] and robotic-assisted mini-invasive surgery (RAMIS) [48,49].

Currently, a new project founded by NASA involving a tiny surgical robot called MIRA (miniaturized in-vivo robotic assistant) [50] is under realization to be tested aboard the ISS in 2024. However, this review is mainly focused on emergency and trauma surgery during long-term space missions and missions to other planets. Robotic surgery can indeed represent the best option during an exploratory space flight far from Earth, as it allows the separation of internal body parts from the external room [51,52], allowing the containment of body fluids inside the abdominal or thoracic cavity. Another advantage is that it prevents contamination of the spacecraft area while, at the same time, protecting internal viscera and solid organs from contact with particles floating sparsely in the spacecraft [52]. It must be underlined, however, that the present surgical robots are teleoperated devices that need the presence of a surgeon at the console.

Through the years, many prototypes of a “space operating room” have been produced [29]. The initial idea was to reproduce in some way the dedicated and selective environment of an operating room on Earth completed by, due to the effects of absence of gravity, the necessary restraint devices for the surgeon, the anaesthetist, or the physician, and the patient [29,52]. However, laparoscopy and then-robotic surgery had to be adapted to the room capacity of the spacecraft. Major issues are the limited room for supplies and storage [29], besides the challenges of space communication with Earth. Some special features differentiate a surgical robot (SR) designed for spacecraft, such as: in that the first must be provided with: extra storage for a dedicated library to be consulted in case of need, the ability to perform some diagnostic procedures, such as ultrasound examination or CT scan, and the capability to preserve and monitor vital functions [29,37]. However, a surgical robot is not to be considered the best option under all conditions. For example, in the case of a severe traumatic event requiring immediate action, be it in space or on Earth, it may be necessary to rely on open surgery. On the other hand, in space, such as in all extreme environments, some procedures commonly utilized on Earth [44], such as angioembolization, are not feasible, at least for now. Besides, open surgery requires an experienced surgeon. If the surgical procedure proves to be ineffective, difficult decisions need to be made, from “mission abort” to discontinuation of treatment to the injured crew member in order to preserve the resources still available for the rest of the crew [44]. New study protocols to be applied in extreme situations shall be necessary, dedicated expressly to space trauma and its treatment [44]. In 2022, an experiment on completing in vivo an anastomosis of porcine intestine with the smart-tissue autonomous robot (STAR) was conducted in a highly controlled setting [53]. Nascent clinical viability of an autonomous soft-tissue surgical robot was demonstrated for the first time [53,54]. STAR was controlled by artificial-intelligence (AI) algorithms, receiving input from an array of visual and haptic sensors [55] that can create an experience of touch by applying forces, vibrations, or motions to the user [55]. Haptic sensors function by applying a combination of force vibration and motion to recreate the sense of touch. They are divided into three systems: cutaneous, kinaesthetic, and haptic [56,57]. Furthermore, the tactile sensation can be divided into “active”, “passive”, and “haptic” sensations; an example of active action is object recognition [58,59]. These technologies may create virtual objects in a computer simulation, but can also enhance remote control of devices (telerobotic). In addition, haptic devices may carry tactile sensors to measure and record forces exerted by the user on the interface [60] and facilitate investigation of how the human sense of touch works by allowing the creation of controlled haptic virtual objects [60] (Table 1).

As conventional surgical robots in our ORs are teleoperated devices controlled in real time by humans, they do not have the capability to autonomously perform surgical tasks [38]. With regard to artificial intelligence, the definition of autonomy is “the ability to perform the intended tasks based on the current state and detection without human intervention”. The International Organization for Standardization (ISO8373:2012) defines “Autonomy” as a gradual progression of various degrees of human intervention, from non-independence to full independence [61].

It is well known that conventional surgical robots are insensitive to fatigue, have tremor-filtering capabilities, scalable motion, and an increased range of axial motion [48]. The possibility of combining AI control algorithms with the inherent peculiarities of surgical robots could help reduce technical errors and operative times, and may allow access to hard-to-reach body areas, improving outcomes [53,54].

According to Panesar 2018 [11], a “clinically capable robot may be able to provide surgical care in environments where care provision is lacking”. An example is aboard a spacecraft in “deep space” or in war zones inaccessible to surgical care, as well as in environmental disasters where structures are severely damaged or unavailable.

A surgical robot capable of autonomous actions is defined through three parameters: mission complexity, environmental difficulty, and independence from human control [61]. Robots in “autonomous mode” should have the ability to see, think, and act through the interactions of visual and physical sensors that perceive the environment without active human intervention to achieve a predetermined surgical goal safely and effectively. Such a result requires a high degree of integration of algorithms, robotics, computer vision, and intelligent sensor technologies, as well as a large and comprehensive trial period [61]. However, as specified above, we are far from these achievements given that for the time being such robots do not even exist. Panesar et al., in their publication in 2019 [11], concluded that it could take until the end of this century before technology may be able to realize them.

**Table 1:** Haptic Sensors—Summary.

What does haptic mean in robotic surgery?	Haptic is an adjective relating to the sense of touch, specifically relating to the perception and manipulation of objects using the senses of touch and prio-prioception. ***“Haptic technology is a technology that can create an experience of touch by applying forces, vibrations or motions to*** [62] ***”***
Short overview	Vision and haptics are the primary senses employed in manipulating objects by humans, but while visual capabilities have reached an advanced stage in robotic surgery, tactile feedback is affected by other challenges such as circuit stability control. The lack of tactile feedback in a teleoperated system, such as that used in operating rooms today, forces the surgeon to depend only on visual cues, increasing the risk of tissue laceration or suture breakage. The goal is to provide the surgeon with information on the strength required for operations so that the operator feels as if she/he is present at the remote site [63]
Terminology	*Touch and somatic senses:* perception is induced by pressure, vibration, skin stretching and temperature. These mechanisms are divided into **cutaneous** or **tactile** and **kinesthetic**, they are linked to the awareness of the position of the person and the movement of the parts of the body through sense organs (proprioceptors) in the muscles and joints [63]. Consequently haptic devices also are categorized as follows: **kinesthetic devices** that generate force/torque feedback usually actuated by electric motors, **tactile displays** that convey contact information to the skin, with small influence on kinesthetic sensation [63]
Applications	On Earth: In minimally invasive surgery (MIS) and robot-assisted minimally invasive surgery (RAMIS) and simulation for surgical training. In Space Research: In Robonaut-2, a humanoid robot in space, [63] and its possible subsequent improvements

### 4.2. Crew and Crew Medical Officer (CMO)

Also, the selection criteria of a crew medical officer (CMO) have been changed through the years [64]. Currently, the target of medical care for the crew in present and future missions is the health, well-being, and safety of the astronauts [63], provided that as the distance from the Earth grows, crew autonomy will have to increase. At this time, a mission on the ISS requires a preflight CMO training of 40–60 h with lectures and practical lessons, medical diagnostics and therapeutic lessons, and cardiopulmonary resuscitation (CPR) and advanced cardiac life support (ACLS) training. The course includes a broad area of medical subjects without a dedicated surgical expertise. In ISS missions, the CMO is, generally, one of the astronauts and not a physician. Two CMOs are considered necessary to serve a six-person crew, but as the level of care increases with the level of the mission, the number of CMOs also increases (according to the so-called need for redundancy). In exploration-class missions, the CMO must be a physician, but additional CMOs are not required to have a medical background. All of them need periodic refresher training both in the prelaunch phase and for the duration of the mission to maintain the required medical ability [64]. In future space missions, the CMO will receive more extensive training than ISS missions’ requirements [65]. Also, a crew member might be trained for the task in case no medical personnel are available for the CMO position. The CMO knowledge for long-duration space missions should also include the treatment for prolonged exposure to the hazards and risks related to space travels [64,66] described in previous sections.

As conditions such as appendicitis, peptic ulcer, or intestinal obstruction cholecystitis, diverticulitis, or trauma of any type and severity [67,68] are classified at the highest level of crew health concern [20,69,70,71], space medical crew selection and training has been extended to achieve more comprehensive surgical capabilities [70,71,72]. The training is designed to specifically instruct the physician, who will be the CMO of the mission, and a medical assistant to help with the task under the CMO supervision and to replace her/him in the case of disease or injury [69,71].

Surgical skills must be maintained for all CMOs, assistants, and crew. A virtual-reality (VR) [73,74] and augmented-reality (AR) [75,76] simulator can be useful in achieving this target [71], as has been demonstrated by experiments on Earth in some surgical disciplines [73,74,75,76,77].

### 4.3. Exploration-Class Missions

Starting from the end of the Apollo program in 1972, many other space missions have been conducted up to the present one; Artemis, begun in 2017, was the first exploration mission to go beyond low orbit (i.e., an orbit at an altitude of between 160 and 1000 km) [77,78].

Life-support systems should be consistent with manned spaceflight, as well as with an adequate provision of appropriate supplies and medical equipment for the estimated health risks [79] in lunar missions. Arrangements may also change based on technological evolution and improvements in spacecraft design. Space medicine needs proper consideration because it reduces the risk of disease and death by treating preventable medical events [79].

In lunar missions, the inability for a “relatively” rapid return to Earth for the wounded or seriously ill, the difficulties in guaranteeing remote medical support, and the limited resources must also be considered [16]. Another task in lunar missions is to evaluate assistance protocols for future explorations of Mars, where returns to Earth, both immediate and deferred, will not be possible [80].

In addition, if no new advances in propulsion technology shall be developed in the meantime, a flight to Mars with a direct return to Earth will last at least nine months or more. During a flight to Mars, delay in communication can extend up to 20 min while in the event of a blackout, the absence of contact with the Earth may last for weeks [80].

**Figure 1:**
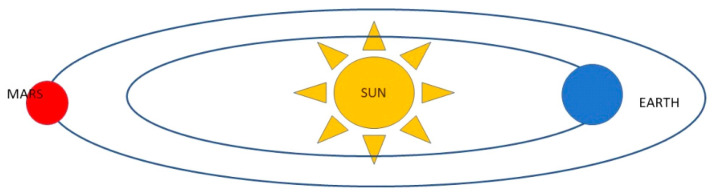
Mars Solar Conjunction—Mars and Earth will be on opposite sides of the Sun. The Sun expels hot, ionized gas from its corona, which extends into space. During solar conjunction, the gas can interfere with radio signals in communication with spacecraft and Mars, resulting in unexpected behaviour. https://www.jpl.nasa.gov/news/whats-mars-solar-conjunction-and-why-does-it-matter (accessed on 20 March 2023).

An inevitable blackout shall occur during the “solar conjunction” (Figure 1) when the spacecraft will not be able to communicate with Earth for about two weeks every two years [81]. This peculiar phenomenon is due to the obscuring caused by the Sun passing between the Earth and Mars, whereby the Earth and Mars cannot “see” each other as the Sun is on the trajectory between the two planets [80]. No attempt can be made to send new instructions to Mars during solar conjunction, as it is impossible to predict the effects of interference from charged particles from the Sun on data transmission which could cause loss of important information. This challenge must be taken into account not only when travelling to Mars but even more so when settling on the planet’s surface [80,81,82].

A NASA report [83,84] identified 30 risks of spaceflight to human health and performance. Among them, the most relevant were found to be: gravity [85] force at the moment of the take-off, propulsion, communications, secondary radiations, nutrition and food, absence of gravity effects in space voyages to Mars and the three-eighths of Earth’s gravitational pull on its surface, mental health, landing, lack of resources, exploration risks, the severe environment, the absence of Earth life conditions on any other planet (atmosphere, for example), and space junk [86,87].

In addition [87], the strategies already developed against most of the risks associated with travel in low Earth orbit need to be further studied, as they are deemed insufficient for long-distance and long-duration missions [82,83].

A 2014 Nasa report [84,87] described the investigations carried out to assess the risks to human health, mostly conducted on Earth and on the International Space Station to outline procedures, drugs, devices, and countermeasures, as well as the possible radiation effects on the human body. It is clear that surgery in space is an immense and expanding field of research, not limited to the use of surgical devices. Although the evolution of surgical robots for space will encompass the application of AI and machine learning, these new achievements cannot cover every possible situation due to the extreme environmental conditions. It is therefore intuitive that the astronauts selected for the first mission on Mars will have to accept an exceptionally higher level of risk [81].

Moreover, conditions during the flight to Mars or the return flight to Earth will be different after having landed and settled on the Red Planet, as compared to what the crew has experienced until now on the ISS. Today, no data are available on the real effects of gravity on Mars (38% of gravity on Earth) [81,85].

Other mission targets are under way, for example the construction of the cislunar space station Gateway (esa.int/Newsroom/Press Release/Exo-Mars starting from 2024) [88]. The Gateway, a vital component of NASA’s Artemis program [87], will serve as a multi-purpose outpost orbiting the Moon, providing essential support for long-term human return to the lunar surface and serving as a staging point for deep-space exploration [89]. In long-duration missions, any improvement in healthcare, be it medical or surgical, will provide crews with new means to cope with the “stay in space” in artificial living conditions offering an acceptable quality of life [90]. These new habitats should also support people’s well-being and a healthy physical and mental state, while minimizing the negative effects of travel and environmental issues in space [90]. Regarding these proposals, NASA has elaborated a “Human Research Program” to organize hazards awaiting astronauts in the first missions to Mars into five main categories: radiation, isolation and confinement, distance from Earth, gravity (or lack of gravity), and hostile/closed environments [90]. Studies have been conducted on ground-based analogues, laboratories, and on the International Space Station (ISS). In addition, NASA has identified and recruited dedicated teams within the agency to study the evolution of these specific areas, with a focus on the next touchdown on Mars [83]. This in-depth knowledge will offer growth opportunities also in innovation, technology, medicine, and in the understanding of the human body’s physiology and pathophysiology. The program includes: information on human health, habitability standards, countermeasures and their development, and risk-mitigation planning, as well as studies conducive to progress and advances in habitability and medical support technologies [90,91,92,93]. Other issues are man-made vessels for long-distance space travel, space platforms for large-scale human habitation, and planetary colonies. New solutions will need to be found, such as effective systems to replenish resources and minimize waste, and an artificial ecosystem specifically designed to ensure long-term support of human life [90,91]. In particular, many authors agree that, in the next decade, enhancement of medical autonomy should be considered a priority (CSA) [91,92,93,94,95]. Other conditions specific to this type of mission, are having to manage limited resources and the need for scientific and technological improvements to ensure greater medical autonomy [90,92,95]. In a paper of 2019, Dutil-Fafard [94] presented a medical condition requirements study funded by the Canadian Space Agency that proposed a scenario-based query capability to support the medical autonomy of astronauts. This publication also refers to a NASA study on high risk of medical events and the need for risk-assessment and decision-support tools [94], with the objective of providing medical infrastructure advances in medical knowledge, skills, and materials improvements. An extensive literature review has been performed and summarized in a medical condition database. The result of these studies has been the identification of the three highest-risk medical conditions to be considered in addition to surgical events: acute coronary syndrome, sepsis, and stroke.

## 5. Discussion

The principal targets of next space missions are:

- build a lunar base;
- explore deep space;
- planning space flights to another planet (Mars);
- plan to settle on another planet (Mars) after a long stay in space.

To date, missions on the ISS are within low Earth orbit (LEO), protected by the Earth’s magnetic shield (magnetosphere) [89]. Going beyond this limit in lunar missions or missions to Mars will expose astronauts to other risks, mainly exposure to space radiation and microgravity, as well as altered gravity, beside the hazards identified by NASA in 2019 [85].

Studies on these topics have important limitations, especially the size of experiments and the amount of collected data. Besides, if some of the studies are reproduced on Earth, investigations are difficult to obtain. However, simulation of blood flow in space was achieved by using bed-rest data from human subjects [95]. Other studies have been performed on the modifications of CO_2_ levels in space [96].

Other systemic and physiological health risks are reported in the literature and deserve to be mentioned in this review: cardiovascular dysregulation, CNS impairments, increased cancer risk, muscle degeneration, bone loss, immune dysfunction, increased liver disease and lipid dysregulation, circadian rhythm dysregulation, and space-associated neuro-ocular syndrome, which have been extensively studied on ISS missions [7,16,20,66,69,97]. However, not much is known about deep-space missions or missions to Mars relatively to the effects of prolonged exposure to the space environment.

New goals for experiments of surgery in space have to be considered. Initially, studies have been conducted on open surgery which evolved to laparoscopic surgery during parabolic flights [33,51]. The general benefits of laparoscopy are temperature control and biological protection of both the crew and the atmosphere from the propagation and floating of bodily fluids in the spaceship, as well as from airborne environmental bacteria [52]. In robotic or mini-invasive surgery, pressurized gas may leak and spread biological contaminants into the atmosphere when changing the instruments through the abdominal ports. As airborne particles do not settle in space, the pressurized contaminated aerosols must be contained to avoid their diffusion in the spacecraft [51,52].

In laparoscopy, effects on the cardiovascular system in a sick or injured astronaut who has already suffered a decrease in blood volume are not yet well known [15,16,35,36]. A decrease in venous return to the heart could lead to visceral hypoperfusion, and also to reduced blood flow to other organs [15,16,35,36]. In addition, other microgravity-induced modifications are present [15], for example, in animal studies [51,52] conducted in parabolic flights, the abdomen was no longer flattened and oval in shape but appeared rounder, displaying an increase in internal volume, allowing easier manipulation of the instruments during the procedure. Abdominal pressure greater than 15 mm Hg on Earth determines hypertension (intra-abdominal hypertension, or IAH), that can shift to severe hypertension and abdominal compartment syndrome (ACS) with reduced ventilatory compliance hypoxemia, hypercarbia, and respiratory failure, associated with cardiac and renal impairment. In weightlessness, in a parabolic flight study [33] of anaesthetized pigs on the IAH, instead of marked ventilatory disturbances, there was an increase in dynamic compliance [33]. In addition, adaptation to microgravity in astronauts in long-duration missions, leads to a peculiar physiologic state not only for blood volume and circulation, but also for neurohumoral alterations implying reduced compensation in the case of hypovolemia and shock [17,35,36]. In addition, the systemic absorption of CO2 leads to hypercarbia, pulmonary hypertension, and acidosis. However, the ability to separate the external environment from the intra-abdominal excludes contamination between the two systems, and prevents the patient from cooling down as occurs in the open abdomen, allowing faster healing.

This technique has naturally evolved toward robotic surgery, with a human operator in control. The robot’s functions are able to improve the overall quality: motion scaling, adaptive tremor filtering, surgical navigation, and augmented reality systems [49,98,99]. In deep-space exploration missions, a robot that includes a human–system interface and a master console composed of a haptic device (see Table 1) for position–orientation input, a video display, and headphones for video and voice feedback, could provide a possible solution. In particular, voice feedback is a device, presently under evaluation, that can recognize the structures from the noise produced during tissue palpation and that has, so far, produced promising results from testing [98,99]. In fact, the ideal solution would also be to have a number of force and tactile sensors to determine the mechanical properties of tissue consistency for more accurate dissections [55,56,57,100].

Recently, versatile robots, such as Robonaut 2 (R2) [101], have been tested for use in medical procedures, but, so far, the robot is not precise enough for surgery. A dexterous humanoid robot that serves as an effective medical and surgical assistant is under study. Many other studies are running, for example, on small scale in-body robots that can enter the abdominal cavity through a small incision or a natural orifice [102,103], or swallowable self-assembling robots that can be controlled externally through magnets [103,104].

Human explorations in space represent a peculiar condition that require research on advanced healthcare development of surgical procedures and diagnostic possibilities. Up to this day, artificial intelligence (AI) [20,53,54] is the most accredited solution for the further evolution of robotic technology in surgery. It is possible that in the future a human-inspired, dexterous robot shall be present on an exploration spacecraft to assist the CMO in many possible tasks. The objective of combining robotic surgery with AI is to increase procedural efficiency, accuracy, and safety, as well as to decrease the surgeon focus on automatable tasks and overall fatigue. However, a huge amount of work is still needed to integrate all the instructions required to introduce a new kind of robotic surgical device onto the spacecraft, to ensure the necessary level of robot autonomy to safely perform surgical tasks, as well as to apply the constraints that need to be imposed on the robot. The application of safety protocols should always guide the use of a robotic assistant. Also, the crew must be trained with the robot pre- and in-flight, but both guidelines and training must also be extended to non-surgical personnel. Any simple action requires different capabilities, for example, computer vision, recognition of the suture thread, and additional software to perform movements to obtain and tie the knot. Surgery is a complex task that requires both technical and decision-making skills but, for now, only small surgical tasks and sub tasks automation are applicable.

In space, a dedicated surgical robot provided with an image-guided autonomous system, ultrasound, magnetic resonance imaging, or computed tomography scans, pre-programmed to perform basic surgical procedures, anaesthesia support, and vital-signs monitoring and post-operative care, is the most desirable [62]. Any improvement and modification promoted by “artificial-intelligence” applications, as well as medical device miniaturization, can also be useful on Earth. The features characterizing robots shall, therefore, be increasingly adapted to the tasks they will be progressively called upon to perform.

Two intrinsic functions should be performed by robots: to accomplish the pre-programmed goals of the procedures; and to be able to dynamically respond to an ever-changing surgical environment [11,49,98,99] in other words, go through the robotic perception mapping to begin with, and then plan efficient actions for the future. However, to mimic a human surgeon’s perception in real time is a much more complex action than simply applying AI algorithms. A robot must be taught how to perform surgery. This is a complex action, peculiar to the human mind, and mimicking a human surgeon effectively requires not only the ability to detect and interpret all relevant sensory inputs and positional information, but also to have an extensive database of explicit knowledge on how to safely proceed to achieve the surgical goal [11,32]. Although we are presently far from the creation of such a robot, some of the improvements and new discoveries stemming from space research, such as the smart robot “Brain Surgeon” [105], the smart-tissue-autonomous robot for tissue or SMART [53,54], “MRI and CAT scans” [106,107], the “Multi-layer Microcapsules Help Drug Delivery/Fight Tumours” [108,109], and others, have many applications that can already improve life on Earth.

## Data Availability

The available information on references and web connections are all published in the article section REFERENCES.
